# Calicivirus from Novel Recovirus Genogroup in Human Diarrhea, Bangladesh

**DOI:** 10.3201/eid1807.120344

**Published:** 2012-07

**Authors:** Saskia L. Smits, Mustafizur Rahman, Claudia M.E. Schapendonk, Marije van Leeuwen, Abu S.G. Faruque, Bart L. Haagmans, Hubert P. Endtz, Albert D.M.E. Osterhaus

**Affiliations:** Erasmus Medical Center, Rotterdam, the Netherlands (S.L. Smits, C.M.E. Schapendonk, B.L. Haagmans, H.P. Endtz, A.D.M.E. Osterhaus);; Viroclinics Biosciences B.V., Rotterdam (S.L. Smits, M. van Leeuwen, A.D.M.E. Osterhaus);; and International Centre for Diarrhoeal Disease Research, Bangladesh, Dhaka, Bangladesh (M. Rahman, A.S.G. Faruque, H.P. Endtz)

**Keywords:** human, calicivirus, metagenomics, Recovirus, random amplification, next-generation sequencing, Bangladesh, novel, viruses, recoviruses, Caliciviridae

## Abstract

To identify unknown human viruses in the enteric tract, we examined 105 stool specimens from patients with diarrhea in Bangladesh. A novel calicivirus was identified in a sample from 1 patient and subsequently found in samples from 5 other patients. Phylogenetic analyses classified this virus within the proposed genus Recovirus.

Diarrhea, characterized by frequent liquid or loose stools, commonly results from gastroenteritis caused by infection with bacteria, parasites, or viruses. Patients with mild diarrhea do not require medical attention; the illness is typically self-limited, and disease symptoms usually resolve quickly. However, diarrheal diseases can result in severe illness and death worldwide and are the second leading cause of death around the world in children <5 years of age, particularly in low- and middle-income countries (*1*). For many cases of diarrhea in humans, no causative agent is identified.

In recent years, many novel viruses have been identified in human and animal blood, respiratory secretions, and fecal material through viral metagenomic studies consisting of random amplification in combination with next-generation sequencing methods (*2**–**5*). To identify unknown human viruses in the enteric tracts of persons with diarrhea, we performed sequence-independent amplification on purified viral nucleic acid from fecal samples obtained from patients with diarrhea in Bangladesh (*6**,**7*). We identified a novel calicivirus and classified it in the proposed genus Recovirus. Caliciviruses, which are nonenveloped, positive-stranded RNA viruses with a polyadenylated genome οf ≈6.4–8.4 kb, cause illness in animals and humans (*8**,**9*), including gastroenteritis in humans. The family *Caliciviridae* consists of 5 genera, *Norovirus*, *Sapovirus*, *Lagovirus*, *Vesivirus*, and *Nebovirus*, and 3 proposed genera, Recovirus, Valovirus, and chicken calicivirus (*8**–**10*).

## The Study

Each year, >100,000 diarrhea patients are admitted to the Dhaka hospital of the International Centre for Diarrheal Disease Research, Bangladesh (ICDDR,B). Fecal samples from 2% of these patients are collected and examined as part of systematic routine surveillance system for the presence of enteric pathogens (*11*). All procedures were performed in compliance with relevant laws and institutional guidelines and in accordance with the Declaration of Helsinki.

Stool specimens from a subset of patients from routine surveillance during 2007–2009 (1,614 samples total) were available for further studies. These specimens were prescreened for adenovirus and rotavirus A by using TaqMan EZ RT-PCR Core Reagents (Applied Biosystems, Foster City, CA, USA), rotavirus primers RVNSP3R and RVNSP3F and probe 5′-FAM-AGTTAAAAGCTAACACTGTCAAA-TAMRA-3′ (*12*), and TaqMan Universal Mastermix (Applied Biosystems) (*13*). Sequence-independent nucleic acid amplification and next-generation sequencing were performed on 105 stool specimens from diarrhea patients enrolled during 2007 by using a 454 GS Junior Instrument (Roche, Indianapolis, IN, USA) as described (*6**,**7*). More than 725,000 trimmed reads were assembled by using de novo assembly and analyzed according to BLAST searches (Technical Appendix Figure 1 and Table 1) (*6**,**7*). Sequences were classified on the basis of the taxonomic origin of the best-hit sequence (*6**,**7*). An E (expect) value of 0.001 was used as cutoff value of significant virus hits. The largest proportion of virus-related sequences in human diarrhea samples from Bangladesh in 2007 was related to known bacteriophages and mammalian viruses (Technical Appendix Figure 1).

**Figure 1:**
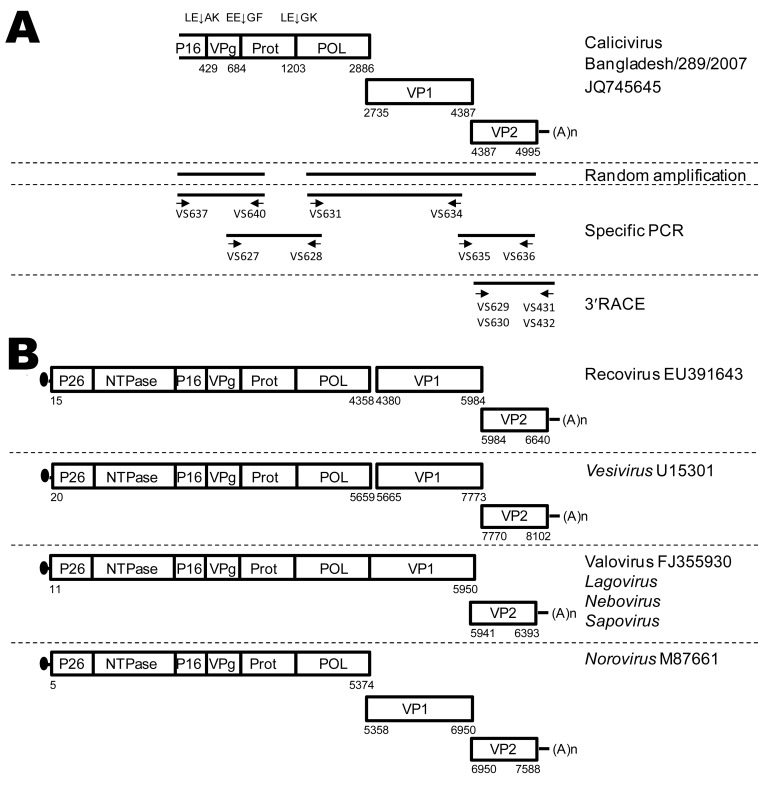
Schematic outline of the strategies used for PCR amplification of calicivirus Bangladesh/289/2007. A) Schematic representation of the calicivirus Bangladesh/289/2007 genome. Boxes represent the open reading frames encoding the calicivirus proteins. Indicated are the poly(A)-tail (A_n_); putative cleavage sites indicated by XX↓XX. The 5′ end of the genome was not obtained. The bottom of the panel shows a schematic outline of the reverse transcription PCRs employed to amplify calicivirus Bangladesh/289/2007 sequences by using random amplification, degenerate PCR, and 3′ rapid amplification of cDNA ends (RACE) PCR. The orientations and positions of the oligonucleotides on the calicivirus genome are depicted and sequences shown in Technical Appendix Table 2. B) Genome organization of caliciviruses in the genera *Vesivirus*, *Nebovirus*, *Norovirus*, *Sapovirus*, and *Lagovirus* and proposed genera Valovirus and Recovirus, for comparison with the new calicivirus Bangladesh/289/2007. The 5′ end of the genome is shown with a Vpg protein (black dots). Numbers indicate the nucleotide positions according to the virus genome for which the GenBank accession number is indicated.

One sample, no. 289, yielded a novel mammalian virus from the family *Caliciviridae* that we further characterized by near full-length genome sequencing using random amplification with next-generation sequencing, specific reverse transcription PCRs, and 3′ rapid amplification of cDNA ends PCR (Figure 1, panel A) as described (*6**,**7*). We named the virus isolate calicivirus Bangladesh/289/2007 (GenBank accession no. JQ745645).

**Figure 2:**
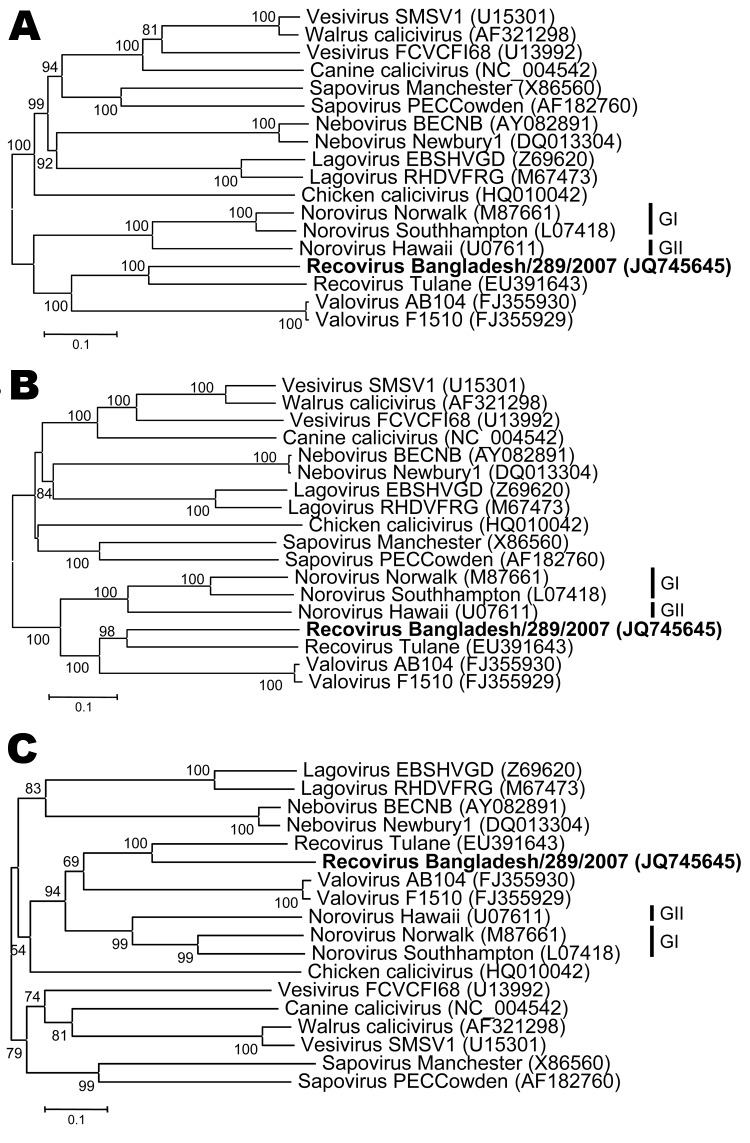
Neighbor-joining phylogenetic trees of the amino acid sequences of the partial polyprotein sequence (A), viral protein (VP) 1 (B), and VP2 (C) capsid proteins of selected representative caliciviruses and the newly identified Recovirus Bangladesh/289/2007 (indicated in **boldface**). Phylograms were generated by using MEGA5 (www.megasoftware.net) with p-distance and 1,000 bootstrap replicates. Significant bootstrap values and GenBank accession numbers are shown. Scale bars indicate amino acid substitutions per site.

The *Caliciviridae* genome encodes a polyprotein precursor for nonstructural proteins, and 2 structural capsid proteins, viral protein (VP) 1 and VP2, from 2 or 3 open reading frames (ORFs) (Figure 1, panel B) (*8**,**9*). The genome organization of Bangladesh/289/2007 is most closely related to that of viruses in the genus *Norovirus*, with ORF2 encoding VP1 overlapping with ORF1 (Figure 1). The partial polyprotein precursor and complete VP1 and VP2 proteins were aligned with corresponding sequences of representative caliciviruses. Divergence analysis demonstrated that the calicivirus Bangladesh/289/2007 showed 57.7%, 49.5%, and 53.1% amino acid identity in the polyprotein, VP1, and VP2, respectively, to its closest relative, Recovirus Tulane (GenBank accession no. EU391643), which was identified from rhesus macaques (Table 1; Figure 2).

**Table 1:** Percentage amino acid identity of calicivirus Bangladesh/289/2007 with representative caliciviruses of other genera*

Calicivirus genus	Strain	GenBank accession no.	NS polyprotein†	VP1	VP2
*Norovirus*	Norwalk	M87661	27.7	30.4	24.7
	Southampton	L07418	29.0	31.2	23.3
*Sapovirus*	Manchester	X86560	20.1	18.6	4.1
	PEC Cowden	AF182760	18.3	18.1	6.8
*Vesivirus*	FCV CFI68	U13992	18.4	16.3	15.1
	SMSV 1	U15301	19.0	19.3	5.5
*Lagovirus*	RHDV FRG	M67473	18.0	19.6	9.6
	EBHSV GD	Z69620	17.3	17.8	5.5
*Nebovirus*	BEC NB	AY082891	18.7	16.8	11.0
	Newbury 1	DQ013304	19.0	16.8	11.0
Recovirus‡	Tulane	EU391643	57.7	49.5	53.1
Valovirus‡	AB104	FJ355930	35.8	43.5	26.0

*NS, nonstructural; VP, viral protein. †All polyprotein sequences were aligned without the capsid protein encoding sequences. ‡Tentative genus not yet accepted by the International Committee on Taxonomy of Viruses.

Additionally, on the basis of the identity profiles of noroviruses (*14*)—strains, genotypes, and genogroups showed 85.9%–100%, 56.2%–85.7%, and 38.6%–55.1% identity, respectively—Bangladesh/289/2007 may represent a new genogroup in the proposed genus Recovirus. This conclusion was corroborated by comparison of a partial RNA-dependent RNA polymerase (RdRp) sequence of calicivirus Bangladesh/289/2007 to known recovirus RdRp sequences (Technical Appendix Figure 2).

We performed a diagnostic real-time recovirus PCR targeting the RdRp of all 1,614 available samples from patients with diarrhea (*11*). Reverse transcription PCR–grade viral nucleic acid was extracted by using the MagNA Pure LC Total Nucleic Acid Isolation Kit (Roche) and amplified by using reverse transcription PCR with primers VS665 and VS666 and probe VS664 (Technical Appendix Table 2) and TaqMan EZ RT-PCR Core Reagents (Applied Biosystems). The cycling program consisted of 50°C for 2 min, 60°C for 30 min, 95°C for 5 min, and 50 cycles of 95°C for 20 s and 59°C for 1 min, resulting in a 164-bp amplicon.

In addition to sample 289, 5 other human diarrhea samples (Table 2) were sequence-confirmed to be positive for a recovirus, with high homology (>98%) to calicivirus Bangladesh/289/2007; this finding indicates that recovirus Bangladesh infects humans. Clinical data indicate that all patients with a recovirus present in their feces had 6 to >21 watery stools in the first 24 hours after illness onset (Table 2); 4 patients experienced vomiting and 2 patients had fever. Patient ages ranged from 3 months to 50 years. Three recovirus-positive patients showed evidence of co-infection with other pathogens that are known to cause diarrhea in humans, such as rotavirus A, adenovirus, *Vibrio cholerae*, or *Salmonella* spp.; the other 3 patients did not. Although viruses such as norovirus, sapovirus, and astrovirus were not detected in the recovirus-positive samples by sequence-independent amplification assays, all samples were not analyzed for all known enteric pathogens. Of 514 diarrhea samples gathered by the Diagnostic Unit, Department of Virology, Erasmus Medical Center, Rotterdam, the Netherlands, for diagnosis of gastrointestinal infections during 2007 and 2009, none was positive for Recovirus Bangladesh (data not shown).

**Table 2:** Clinical data for patients positive for Recovirus Bangladesh/289/2007, Bangladesh*

Patient no.	Age, y/sex	Year of sample collection	Fever	Disease duration, d	Bowel movements†	Vomiting†	Abdominal pain	Patient condition	Other pathogens‡
201	23/M	2007	Yes	<1	11–15	<10	Yes	Lethargic	None
289§	25/M	2007	No	<1	6–10	<10	Yes	Lethargic	None
445	20/M	2007	No	1–3	11–15	0	Yes	Normal	None
507	50/F	2007	No	<1	6–10	<10	No	Drowsy	*Vibrio cholerae*
809	0/M	2008	No	4–6	>21	<10	No	Lethargic	Rotavirus A
1084	1/M	2008	Yes	1–3	6–10	0	Yes	Normal	Adenovirus/ *Salmonella* spp.

*All patients had watery diarrhea. †Number of events in 24 h before sample collection. ‡The pathogens that were tested for were *Shigella* spp., *Aeromonas* spp., *Vibrio cholerae*, *Campylobacter* spp., and *Salmonella* spp. In addition, the samples were screened for *Giardia lamblia*, *Entamoeba histolytica*, *Cryptosporidium* spp., *Ascaris lumbricoides*, *Cyclospora cayetanensis*, *Isospora belli*, adenoviruses, and group A rotaviruses. §The virus from patient 289 is the initially described calicivirus Bangladesh/289/2007 from this study.

## Conclusions

For a large proportion of human diarrhea cases, no etiologic agent can be identified, despite multiple metagenomic studies of viruses in human stool aimed at identifying new etiologic agents (3–5). In addition, it cannot be known when and where emerging and reemerging viruses will appear in the human population. To identify potential etiologic agents of diarrhea in humans, we performed a metagenomic viral inventory in diarrhea samples from Bangladesh, which led to the identification of a novel calicivirus.

Although no species demarcation criteria have been defined for the family *Caliciviridae* by the International Committee on the Taxonomy of Viruses, we classified calicivirus Bangladesh/289/2007 in the proposed genus Recovirus, primarily on the basis of phylogenetic analyses (*8*). Bangladesh/289/2007 may also be considered a new genogroup of the genus Recovirus for the following reasons: 1) the genetic distance between Recovirus Tulane and Bangladesh/289/2007 VP1 is similar to that of VP1 capsids of noroviruses belonging to different genogroups (*14*); 2) the genetic distances between macaque recoviruses and Recovirus Bangladesh/289/2007 RdRP are similar to that of RdRP sequences of recoviruses belonging to different genogroups (*15*); 3) the genome organization of Recovirus Tulane and Bangladesh/289/2007 differs; and 4) Recovirus Tulane and Bangladesh/289/2007 were identified in different host species.

In conclusion, this identification of a novel calicivirus, classified as Recovirus Bangladesh/289/2007, from human diarrhea samples provides PCR-based evidence that recoviruses can infect humans. A previous study found high prevalence of virus-neutralizing antibodies against a closely related calicivirus, Recovirus Tulane, in serum samples from animal caretakers (*15*). Larger epidemiologic studies using genetic and serologic screening will be necessary to provide more insight into the distribution and pathogenic potential of recoviruses in humans.

## Supplementary Material

Technical Appendix Distribution of the percentage of sequence read matches and phylogenetic tree of the nucleotide sequences of the partial RdRP sequence of selected representative recoviruses and the newly identified recovirus Bangladesh/289/2007.
