# Supplementary material for: Calicivirus from Novel Recovirus Genogroup in Human Diarrhea, Bangladesh
Source: Emerg Infect Dis. 2012 Jul;18(7):1192–5. doi: 10.3201/eid1807.120344 (PMC3376821; doi:10.3201/eid1807.120344)
Supplement: Technical Appendix — Distribution of the percentage of sequence read matches and phylogenetic tree of the nucleotide sequences of the partial RdRP sequence of selected representative recoviruses and the newly identified recovirus Bangladesh/289/2007. [file 12-0344-Techapp_3p.pdf]

# Detection of Human Calicivirus in Novel Recovirus Genogroup, Bangladesh

## Technical Appendix

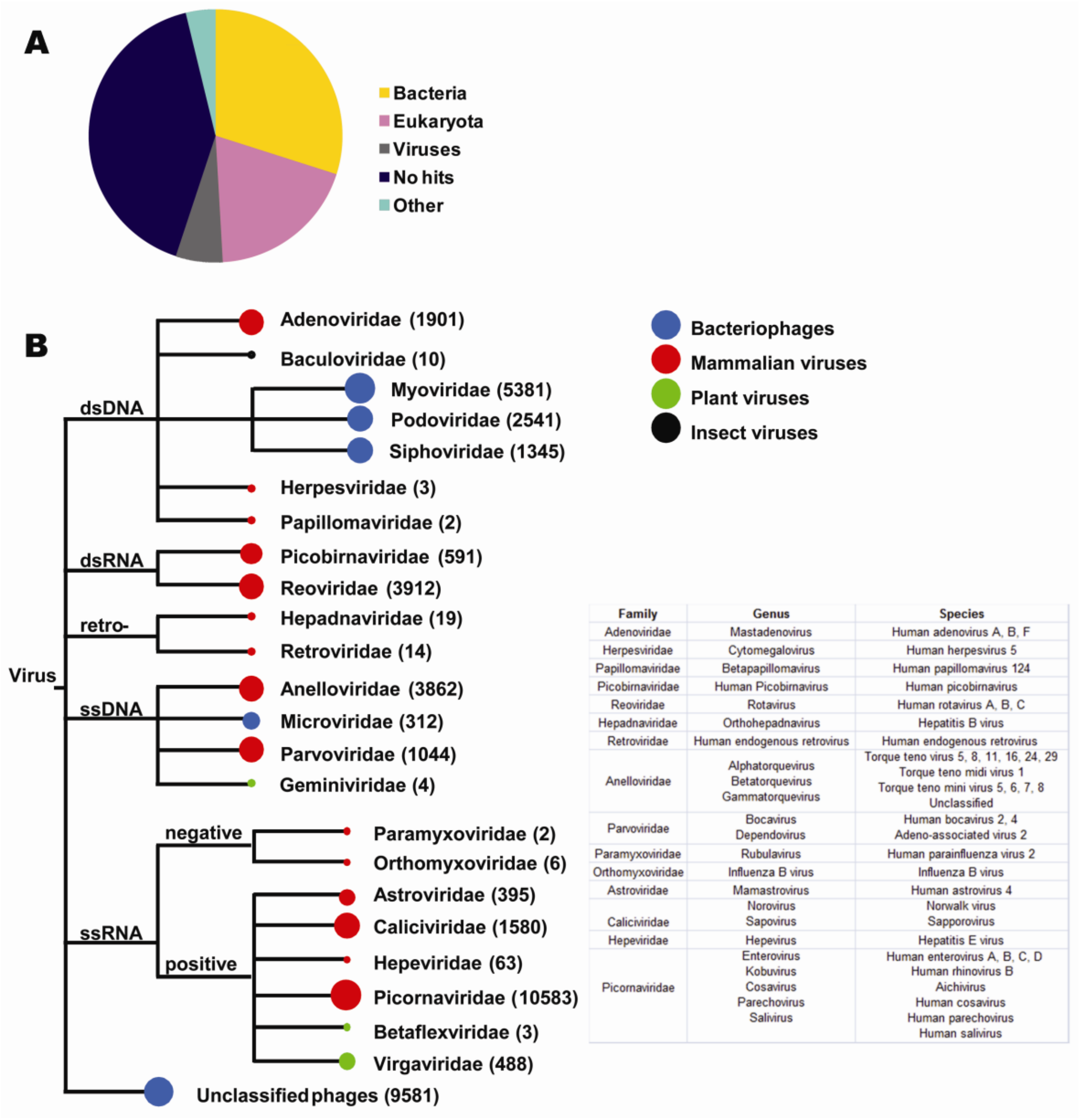


| Family | Genus | Species |
|---|---|---|
| Adenoviridae | Mastadenovirus | Human adenovirus A, B, F |
| Herpesviridae | Cytomegalovirus | Human herpesvirus 5 |
| Papillomaviridae | Betapapillomavirus | Human papillomavirus 124 |
| Picobirnaviridae | Human Picobirnavirus | Human picobirnavirus |
| Reoviridae | Rotavirus | Human rotavirus A, B, C |
| Hepadnaviridae | Orthohepadnavirus | Hepatitis B virus |
| Retroviridae | Human endogenous retrovirus | Human endogenous retrovirus |
| Anelloviridae | Alphatorquevirus<br>Betatorquevirus<br>Gammatorquevirus | Torque teno virus 5, 8, 11, 16, 24, 29<br>Torque teno midi virus 1<br>Torque teno mini virus 5, 6, 7, 8<br>Unclassified |
| Parvoviridae | Bocavirus<br>Dependovirus | Human bocavirus 2, 4<br>Adeno-associated virus 2 |
| Paramyxoviridae | Rubulavirus | Human parainfluenza virus 2 |
| Orthomyxoviridae | Influenza B virus | Influenza B virus |
| Astroviridae | Mamastrovirus | Human astrovirus 4 |
| Caliciviridae | Norovirus<br>Sapovirus | Norwalk virus<br>Sapporovirus |
| Hepeviridae | Hepevirus | Hepatitis E virus |
| Picornaviridae | Enterovirus<br>Kobuvirus<br>Cosavirus<br>Parechovirus<br>Salivirus | Human enterovirus A, B, C, D<br>Human rhinovirus B<br>Aichivirus<br>Human cosavirus<br>Human parechovirus<br>Human salivirus |

Technical Appendix Figure 1. Distribution of the percentage of sequence read matches. (A) The 725305 sequence reads were assembled in 78676 contigs and 156008 single reads, which were analyzed using BLASTn searches. The contigs and single reads were classified based on the taxonomic origin in the annotation of best hit sequence (E-score $<10^{-3}$) as being of bacterial, eukaryotic, other or viral origin and

depicted in a pie chart colored according to the key. The percentage of each category is based on the number of reads per category. A large percentage of reads showed no hits with any sequence in the Genbank database. (B) Taxonomic classification of sequences with similarity to known viruses. Circles located next to taxa are proportional to the total number of sequence reads identified in the indicated category of viruses with BLASTn ($E<10^{-3}$). The number of sequence reads is included in brackets and the different colors indicate whether the virus infects mammalians, plants, insects or bacteria. The table indicates which virus species were identified.

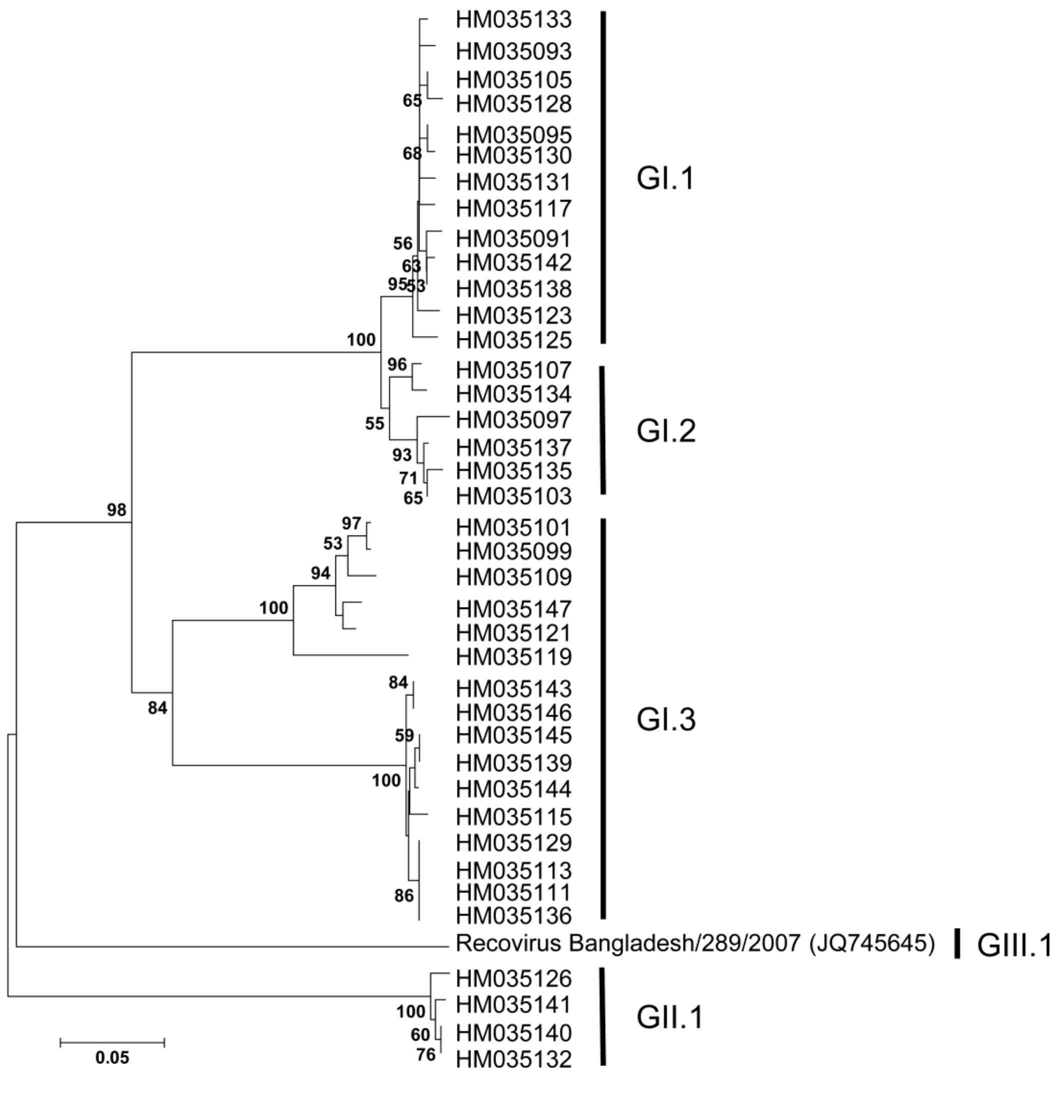


Technical Appendix Figure 2. Phylogenetic tree of the nucleotide sequences of the partial RdRP sequence of selected representative recoviruses and the newly identified recovirus Bangladesh/289/2007. Phylograms were generated using MEGA5, with the Neighbor-joining method with p-distance and 1,000 bootstrap replicates. Significant bootstrap values and Genbank accession numbers are shown, as well as genogroups and genotypes.

Technical Appendix Table 1. Overview of sequence reads and contigs

| No. samples | Read data | | | | | No. contigs | Average no. reads/sample |
|---|---|---|---|---|---|---|---|
| | Total no. | Average length, nt | No. assembled | No. unassembled | % Assembled | | |
| 105 | 725305 | 222 | 569297 | 156008 | 78.5 | 78676 | 6908 |

Technical Appendix Table 2. Oligonucleotides primers

| Primer | Nucleotide sequence, 5′ → 3′ | Orientation | Position* |
|---|---|---|---|
| VS431 | GCGAGCACAGAATTAATACGACT | Reverse | Not applicable |
| VS432 | CGCGGATCCGAATTAATACGACTCACTATAGG | Reverse | Not applicable |
| VS627 | GGAGGGAAGATGCTG | Forward | 527–543 |
| VS628 | GACAGGTTCGTCCTTGAG | Reverse | 1642–1659 |
| VS629 | GCCGCTTTCGCGACTGGTC | Forward | 4396–4414 |
| VS630 | GACTGGTCTAGGCCAAGCTTC | Forward | 4407–4427 |
| VS631 | CAATTATACCAAGAAAGATC | Forward | 1530–1549 |
| VS634 | CCAGAACCAGGAAGAATC | Reverse | 4285–4302 |
| VS635 | CTATAGTATGTATGGTGG | Forward | 4195–4212 |
| VS636 | GTGAGCTAAGAGAAGAC | Reverse | 4905–4921 |
| VS637 | CAGGACCTGAGAGATCTG | Forward | −2 to 16 |
| VS640 | CCAGCTGTGTGTATTGC | Reverse | 1129–1145 |
| VS664 | FAM-CACTGGATTGCCTCAAC-TAMRA | Forward | 2077–2093 |
| VS665 | CAAGGTGATTGTGAATGACCAACT | Forward | 1989–2012 |
| VS666 | CGATTGGCCAGTTTACAGC | Reverse | 2134–2152 |

*Position on genome of calicivirus Bangladesh/289/2007 (GenBank accession no. JQ745645).
